# Emotional exhaustion in nursing students. A multicenter study

**DOI:** 10.1590/1980-220X-REEUSP-2022-0319en

**Published:** 2023-05-12

**Authors:** Ximena Osorio-Spuler, Mónica Illesca-Pretty, Luis Gonzalez-Osorio, Olga Masot, Concepció Fuentes-Pumarola, Silvia Reverté-Villarroya, Laura Ortega, Carolina Rascón-Hernán

**Affiliations:** 1 Universidad de La Frontera, Facultad de Medicina, Departamento de Enfermería, Temuco, Chile.; 2 Universidad de La Frontera, Facultad de Medicina, Departamento de Medicina Interna, Temuco, Chile.; 3 Universidad de Lleida, Departamento de Enfermería y Fisioterapia, Lleida, España.; 4 Universidad de Girona, Facultad de Enfermería, Departamento de Enfermería, Girona, España.; 5 Universidad Rovira I Virgili, Facultad de Enfermería, Departamento de Enfermería, Tortosa, España.; 6 Universidad Rovira I Virgili, Facultad de Enfermería, Departamento de Enfermería, Tarragona, España.

**Keywords:** Students, Nursing, Multicenter Study, Stress, Psychological, Education, Nursing, Estudantes de Enfermagem, Estudo Multicêntrico, Estresse Psicológico, Educação em Enfermagem, Estudiantes de Enfermería, Estudio Multicéntrico, Estrés Psicológico, Educación en Enfermería

## Abstract

**Objective:**

To know emotional exhaustion in nursing students from four universities.

**Method:**

Cross-sectional, correlational study, carried out in Chile and Spain (2017-2018), with 1,368 students answering a self-applied instrument (sociodemographic/academic variables and the Emotional Exhaustion scale). Analysis with Stata 15, according to variables: Chi^2^ tests, Wilcoxon rank sum test (Mann Whitney U test), analysis of variance and multiple regression; confidence level 95% and significance 5% (p < 0.05). Approved by the Ethics Committee, Universidad de Girona.

**Results:**

Academic variables and perceived stress with Quite Much/Much classification: Exams, Problem-Based Learning, Laboratory/Simulation. Statistically significant differences in emotional exhaustion, according to sex, dependent people, workers, commuting time >30 minutes. Greater emotional exhaustion when taking courses for the second time and in academic activities where they declare perceived stress as Quite Much/Much (p < 0.005).

**Conclusion:**

All students present mean level of emotional exhaustion (>26 and <37 points). The variables sex and having dependents are relevant aspects. Stress perceived by methodologies is significantly related to levels of emotional exhaustion.

## RESUMEN

**Objetivo:**

Conocer cansancio emocional en estudiantes de enfermería de cuatro Universidades.

**Método:**

Estudio de corte transversal, correlacional, realizado en Chile y España (2017-2018). Respondieron instrumento autoaplicado 1368 estudiantes (variables sociodemográficas/académicas y escala de Cansancio Emocional). Análisis con Stata 15, según variables: Pruebas de Chi^2^, sumas y rangos de Wilcoxon (Mann Whitney), análisis de varianza y regresión múltiple; nivel de confianza 95% y significancia 5% (p < 0,05). Aprobado por Comité de Ética, Universidad de Girona.

**Resultados:**

Variables académicas y estrés percibido destacan Bastante/Mucho para: Exámenes, Aprendizaje Basado en Problemas, Laboratorio/Simulación. Diferencias estadísticamente significativas entre cansancio emocional según sexo, personas a cargo, trabajadores, tiempo traslado > a 30 minutos. Mayor cansancio emocional al cursar asignaturas por segunda vez y en actividades académicas donde declaran un estrés percibido como Bastante/Mucho (p < 0,005).

**Conclusión:**

Todos los estudiantes presentan cansancio emocional medio (>26 y <37 puntos). Las variables sexo y tener personas a cargo son aspectos relevantes. Estrés percibido por metodologías se relaciona de manera importante con niveles de cansancio emocional.

## RESUMO

**Objetivo:**

Conhecer a fadiga emocional em estudantes de enfermagem de quatro universidades.

**Método:**

Estudo transversal, correlacional, realizado no Chile e na Espanha (2017-2018). Um total de 1.368 alunos respondeu a um instrumento autoaplicável (variáveis sociodemográficas/acadêmicas e Escala de Fadiga Emocional). Análise com Stata 15, de acordo com as variáveis: testes de Chi^2^, teste dos postos sinalizados de Wilcoxon (Mann Whitney), análise de variância e regressão múltipla; nível de confiança de 95% e significância de 5% (p < 0,05). Aprovado pelo Comitê de Ética da Universidade de Girona.

**Resultados:**

Variáveis acadêmicas e estresse percebido destaca Bastante/Muito: Exames, Aprendizagem Baseada em Problemas, Laboratório/Simulação. Diferenças estatisticamente significativas entre fadiga emocional, de acordo com o sexo, dependentes, trabalhadores, tempo de viagem > 30 minutos. Maior fadiga emocional ao cursar disciplinas pela segunda vez e em atividades acadêmicas em que declaram um estresse percebido como Bastante/Muito (p < 0,005).

**Conclusão:**

Todos os alunos apresentam fadiga emocional média (>26 e <37 pontos). As variáveis sexo, ter dependentes são aspectos relevantes. O estresse percebido pelas metodologias está significativamente relacionado com os níveis de fadiga emocional.

## INTRODUCTION

As a result of joint work between the Universidad de La Frontera (UFRO) in Chile and the Catalan Universities in Spain (Girona (UdG), Lleida (UdL) and Rovira i Virgili (URV)), it has been observed that the study plans for training nurses are similar in content and methodological strategies, including clinical experiences that are one of the main tasks for the acquisition of disciplinary and generic skills for care management^(1)^. Despite the similarity, there are particularities in each institution, with regard to the educational model, teacher training, establishment of student-centered methodological strategies, and supervision of clinical practices, since in Catalan Universities they are carried out by nursing assistants whose employment contract is primarily from the health center, called associate professors, while those from UFRO are nurses hired by the institution.

In addition, through empirical observation, teachers of different curricular levels have expressed that although students begin their studies with great enthusiasm, as time passes by, disengagement from obligations, fatigue, feeling of emptiness or failure, low self-esteem, lack of concentration and desire to drop out all emerge, which if prolonged and frequent, trigger high levels of stress and tension that can lead to Burnout syndrome^(2)^, with personal and academic consequences. In the former ones, the progressive development of symptoms of anxiety and depression is perceived, facilitating the appearance of reactions of emotional and cognitive distancing from the activity as a way of coping with the overload that it entails and, in the academic settings, a decrease in performance could be observed^(3)^, which would cause dissatisfaction with the studies, and finally, abandonment^(4)^.

Academic stress has been addressed by researchers in student populations, using various terms. In this study, the term used will be “emotional exhaustion”, defined as the product of an inefficient coping by the university student in the face of adverse events in the settings of his academic life, or due to failures in the regulation of emotional expression^(3)^. The consequences are related to the progressive loss of energy, weakening, overwhelming physical and psychological exhaustion, fatigue, lack of motivation and expression of being tired of studying, cynical attitude, and little commitment to academic work^(5)^.

To understand emotional exhaustion in healthcare students, academic (tests and evaluations, fear of failure in the undergraduate course, workload problems, etc.), clinical (work, fear of making mistakes, negative responses to patients’ death or suffering, etc.), and personal/social (economic problems, imbalance between homework/school work, etc.) conditions shall be considered^(6)^.

There is evidence of the direct relationship of emotional exhaustion with clinical syndromes such as anxiety and depression^(7,8)^, also with its inverse association with academic self-efficacy^(7,9)^, with self-esteem, and difficulties regulating emotions^(10)^. The most frequent behavioral responses are: impaired performance, tendency to cause controversy, isolation, lack of enthusiasm, smoking, alcohol or other drug use, absenteeism, risk of accidents, nervousness, increased or decreased appetite, and increased or decreased sleep. Regarding the psychological responses, the following are highlighted: restlessness, depression, anxiety, disturbance, inability to concentrate, irritability, loss of self-confidence, worries, difficulty making decisions, recurring thoughts and distractibility^(11,12)^.

In university students, emotional exhaustion is usually higher in women than in men, which can be explained by specific psychosocial traits^(13,14)^. In the case of nurses, the evidence shows that they are mostly women, who, unlike men, experience a higher degree of perceived stress in situations related to emotions^(15)^. In relation to the distribution of academic burnout and its indicators based on sociodemographic variables, it can be seen that in the age range between 21 and 23 years, being a woman, not having children and being single appear more critical at the time of presenting the syndrome, being essentially between “mild” and “moderate”^(16)^; economic and family demands are stress factors^(17)^ and exhaustion is significantly associated with self-esteem^(10,18)^. For the academic variables, it can be seen that students in a “delayed” curricular situation and who are at the “first” and “fifth” semesters are more vulnerable to fatigue^(16)^. In other studies, academic situations perceived as stressful are “faculty methodological deficiencies”, “exams” and “public interventions”, which can contribute to students dropping out of school,^(19–21)^ and to be exposed to significant intimidation and harassment^(22,23)^.

Moreover, clinical simulation causes a level of stress between moderate and high. However, despite of this, it allows learning^(24)^. Clinical practices, in complex and changing environments, are additional sources of stress^(15,25,26)^. Furthermore, the worries about evaluation are an element influencing emotional exhaustion^(27)^, besides exhaustion due to the number of academic tasks^(11)^, and the higher the level of stress, the greater the use of substances^(20,28)^.

Considering what has been observed and keeping in mind that the integral formation of the university student is an idea and an attitude that must be achieved and taken into account as a basic principle of the university task^(29)^, and that it should be a fundamental pillar in academic and personal development, to provide better support to students and transform the university into a health scenario, a multicenter study aiming at getting to know the emotional exhaustion in nursing students from a Latin American University (UFRO), and three Catalan ones (UdL, URV, UdG), is proposed; all of them are state universities and have an Institutional agreement. The objectives are oriented to: “characterize the population under study sociodemographically” and “relate academic, sociodemographic variables and level of stress with emotional exhaustion among students from participating universities”, hypothesizing that women, who have dependents and who work, present greater emotional exhaustion, as do those who learn with student-centered methodological strategies.

## METHODS

### Design of Study

Cross-sectional, correlational study whose interest was to observe a group of students at a given moment to measure the level of emotional exhaustion and its possible relationship with sociodemographic and academic variables, without the intervention of the researchers. Multicenter study, carried out in Chile and Spain between the last months of 2017 and the beginning of 2018, according to the academic semesters of each institution. The “STrengthening the Reporting of Observational studies in Epidemiology” (STROBE) checklist was applied to write the article.

### Population

It consists of 2,053 students enrolled in all undergraduate nursing levels from three Catalan (Spain) and one Chilean Universities.

### Selection Criteria

To be a regular student of the career of each Study Center and prior signature of the informed consent.

### Sample Definition

Non-probabilistic, where the students were not chosen randomly. The 2,053 students were invited, with the instrument and informed consent being sent twice through the QuestionPro software whose access link was sent by institutional email, and a period of three weeks being given for the response. At the end of this process, the sample consisted of 1,368 participants, who agreed to participate in the study, answering the instruments, without knowing the reasons of those who did not.

### Data Collection

It was carried out using a self-applied, anonymous, voluntary instrument, reported by QuestionPro software, which consisted of two parts, which did not require answers for all the items as a condition to continue advancing:

Sociodemographic and academic variables: this part of the instrument was designed by the researchers, ensuring content validity, with a review through expert judgment, which allowed assessing its relevance and coherence. The variables considered were: age, sex, marital status, number of children and other dependents, financing system, country of origin, place of habitual residence and residence during the study, work parallel to training, daily commuting time to the university, university of study, course where there are more credits enrolled, methodological strategies, clinical practices. Age was transformed into a categorical variable, generating four subgroups. The commuting time variable was dichotomized in the regression analysis, considering the statistical significance presented. The variables describing perception of stress in academic activities were dichotomously worked, recoding “None” or “Little” in one: “N/L” and the answers “Quite Much” or “Much” in another: “QM/M”. This decision was made to facilitate the analysis, since the interpretation in relation to the negative or positive perception of the perceived stress in the mentioned activities is improved.

Emotional Exhaustion Scale (EES) in university students^(30)^ is unidimensional, with a one-factor structure that explains 40% of the variance and high internal reliability, with a Cronbach’s alpha coefficient of 0.83, consisting of 10 items on a scale of 1 to 5 (rarely – few times – sometimes – frequently – always), which evaluates situations related to the exhaustion factor during the last twelve months of student life. The variable is quantitative, where the score ranges from a minimum of 10 points to a maximum of 50. The following scores were used as criteria to define emotional exhaustion, according to percentiles 25 (26 points), 50 (32 points) and 75 (37 points): less than or equal to 26 points, low exhaustion; greater than 26 and less than 37, average exhaustion; and greater than or equal to 37 points, high emotional exhaustion (Variance = 63.65; Skewness = –0.11; Kurtosis = 2.64).

### Data Analysis and Processing

It was made using the software Stata 15. Prior to everything, a reliability analysis of the EES instrument was carried out, obtaining a Cronbach’s alpha of 0.83, as described by the authors^(30)^. The qualitative variables were expressed with frequencies and percentages, and the quantitative ones with means and standard deviations. The Chi^2^ test was carried out to compare the frequency distribution according to categories, the Wilcoxon rank sum test (Mann Whitney U test) to establish differences in the EES level according to dichotomous variables; analysis of variance to compare EES results according to universities and multiple regression analysis to evaluate the effect of variables of interest on EES. Even when the sample is not random, which implies one of the main sources of bias for an inferential analysis through the analysis of a regression model, the results will only be used for the sample under study. In this model, its validation was verified by normality analysis of residuals, homoscedasticity test and Shapiro Wilk test (p = 0.95). For all statistical analysis, a confidence level of 95% and a significance level of 5% (p < 0.05) were adopted.

### Ethical Aspects

Approved by the Ethics and Biosafety Committee of the Universidad de Girona, Spain, the entity responsible for ethical approval (CEBRU0015-2019 code 07/2019).

The study was self-financed by the research groups from the different participating universities, with no conflict of interest.

## RESULTS

A total of 67% (1368) of the nursing students completed the questionnaire: UdL, 323 (23.6%); URV, 495 (36.2%); UdG, 387 (28.3%), and UFRO, 163 (11.9%), whose results are presented in the following tables, where the variation of the number of data related to the total sample is due to missing data, determined by the non-response of students in the respective items or variables.

It should be noted that in the 4 universities the predominance is of female sex, single people, without dependents or work, with a significant difference in this variable when comparing UFRO with the total group. At the same time, most participants are enrolled for the disciples for first time and have had contact with clinical practices (Table 1).

**Table 1:** Frequency distribution of sociodemographic and academic variables of nursing students from the four universities. – UFRO-Temuco (Chile), UdL-Lleida (Spain), URV-Tarragona-Tortosa (Spain), UdG-Girona (Spain), 2017-2018.

		UdL		URV		UdG		UFRO		Total	
		n	%	n	%	n	%	n	%	n	%
**Sex**	Female	255	81.5	401	84.6	335	86.8	126	78.7	1117	83.8
	Male	58	18.5	73	15.4	51	13.2	34	21.3	216	16.2
Age*	≤ 20	99	31.1	140	28.7	196	51.2	58	35.8	493	36.5
	21–24	166	52.2	256	33.9	130	33.9	91	56.2	693	47.6
	25–28	32	10.1	51	9.4	36	9.4	8	4.94	127	9.4
	>29	21	6.6	41	8.4	21	5.5	5	3.1	88	6.51
**Marital status**	Single	279	86.9	409	84.5	336	87.5	156	95.7	1180	87.3
	Married	7	2.1	16	3.3	9	2.3	2	1.2	34	2.5
	Common-law marriage	32	10.0	57	11.8	38	9.9	4	2.5	131	9.7
	Divorced	3	0.9	2	0.4	1	0.3	1	0.6	7	0.5
**Dependents**	If any	5	1.6	12	2.5	1	0.3	3	1.9	21	1.6
	Do not have	315	98.4	469	97.5	380	99.7	158	98.1	1322	98.4
**Works**	Yes	112	34.8	126	25.6	163	42.5	32	19.7	433	31.8
	No	210	65.2	367	74.4	221	57.5	130	80.3	928	68.2
**Commuting time**	≤30 minutes	281	90.1	416	86.3	261	69.4	105	64.4	1063	79.7
	>30	31	9.9	66	13.7	115	30.6	58	35.6	270	20.3
**Financing system**	Own	56	17.4	82	16.6	99	25.8	10	6.1	247	18.1
	Family	222	68.7	339	68.8	218	56.8	67	41.4	846	62.1
	Mixed	45	13.9	72	14.6	67	17.5	85	52.5	269	19.8
Scholarship*	Yes	153	47.4	276	55.9	207	53.6	103	63.6	739	54.1
	No	170	52.6	218	44.1	179	46.4	59	36.4	626	45.9
Students per Course*	First	91	28.5	167	34.0	123	31.8	43	26.7	424	31.2
	Second	92	28.9	188	38.3	98	25.3	26	16.1	404	29.8
	Third	83	26.0	96	19.6	113	29.2	74	46.0	366	26.9
	Fourth	53	16.6	40	8.1	53	13.7	18	11.2	164	12.1
Disciplines enrolled for the second time*	Yes	53	17.0	122	27.1	68	18.5	24	16.7	267	21.0
	No	259	83.0	329	72.9	299	81.5	120	83.3	1007	79.0
Contact clinical practices*	Yes	315	98.1	357	73.0	264	68.2	152	93.3	1088	80.0
	No	6	1.9	132	27.0	123	31.8	11	6.7	272	20.0

*: p < 0.005 Chi^2^ test. Source: elaborated by the authors.

A significant difference is observed in the master class, seminars, highlighting the PBL with a total of 62.2% in the Quite Much/Much rank, while UFRO (42.9) and UdL (46.5) present lower values. Also in Laboratory/Simulation, there is a significant difference where the total stress in the Quite Much/Much rank is 52.3%, a similar situation occurring with clinical practices. Another academic activity that produces Quite Much/Much stress are exams with 93.1% (Table 2).

**Table 2:** Stress perceived by nursing students in academic activities at each university – UFRO-Temuco (Chile), UdL-Lleida (Spain), URV-Tarragona-Tortosa (Spain), UdG-Girona (Spain), 2017-2018.

Academic activities	Perceived stress level	UdL		URV		UdG		UFRO		Total	
		n	%	n	%	n	%	n	%	n	%
Master class*	N/L	258	80.4	404	81.8	288	74.4	150	92.6	1100	80.6
	QM/M	63	19.6	90	18.2	99	25.6	12	7.4	264	19.4
Seminar*	N/L	132	41.0	336	68.2	218	56.3	96	61.5	782	57.6
	QM/M	190	59.0	157	31.8	169	43.7	60	38.5	576	42.4
PBL*	N/L	129	53.5	183	37.1	80	20.7	92	57.1	484	37.8
	QM/M	112	46.5	310	62.9	306	79.3	69	42.9	797	62.2
Laboratory/simulation*	N/L	78	24.4	312	63.4	156	41.5	98	60.9	644	47.7
	QM/M	242	75.6	180	36.6	220	58.5	63	39.1	705	52.3
Clinical practices*	N/L	167	52.2	216	45.6	188	53.3	25	15.6	596	45.6
	QM/M	153	47.8	258	54.4	165	46.7	135	84.4	711	54.4
Team work*	N/L	176	54.5	201	40.7	185	47.8	85	52.5	647	47.4
	QM/M	147	45.5	293	59.3	202	52.2	77	47.5	719	52.6
Exams/tests*	N/L	13	4.0	35	7.1	20	5.2	26	16.1	94	6.9
	QM/M	310	96.0	459	92.9	365	94.8	136	83.9	1270	93.1
Written work*	N/L	103	32.0	158	32.1	140	36.2	84	51.8	485	35.6
	QM/M	219	68.0	335	67.9	247	63.8	78	48.2	879	64.4
**Oral presentation**	N/L	74	23.0	90	18.2	80	20.7	31	19.1	275	20.2
	QM/M	248	77.0	404	81.8	306	79.3	131	80.9	1089	79.8

*: p < 0.001 Wilcoxon rank sum test (Mann Whitney U test). Source: elaborated by the authors. N/L: None/Little; QM/M: Quite Much/Much.

It is observed that there are statistically significant differences in emotional exhaustion among UdG, UdL and URV and among UFRO, UdL and UdG (Table 3).

**Table 3:** EES score and differences according to the University – UFRO-Temuco (Chile), UdL-Lleida (Spain), URV-Tarragona-Tortosa (Spain), UdG-Girona (Spain), 2017-2018.

	EES score			Difference among means		
University	Mean score	SD	Absolute frequency	UdL	URV	UdG
**UdL**	30.3	8.1	316			
**URV**	30.6	7.9	485	0.35 (p = 1.00)		
**UdG**	32.3	8.1	369	2.07 (p = 0.004)	1.7 (p = 0.011)	
**UFRO**	32.5	7.8	155	2.23 (p = 0.025)	1.9 (p = 0.061)	0.16 (p = 1.00)
**Total**	**31.2**	**8.0**	**1325**			

ANOVA test. Source: elaborated by the authors.

Regarding the sociodemographic variables and their relationship with emotional exhaustion, it is observed that there is a statistically significant difference and the EES score is higher in women, in students who have dependents, who work, who have a scholarship and have commuting time higher than 30 minutes. Regarding the academic variables, those who have taken disciplines for the second time show greater emotional exhaustion. In all academic activities that students report perceived stress as Quite Much/Much, it is observed that they have greater emotional exhaustion (p < 0.005) (Table 4).

**Table 4:** Level of emotional exhaustion according to sociodemographic and academic variables and according to perceived stress in academic activities, of the four Universities (global) – UFRO-Temuco (Chile), UdL-Lleida (Spain), URV-Tarragona-Tortosa (Spain), UdG-Girona (Spain), 2017-2018.

		Global emotional exhaustion level		
Sociodemographic variables	Category	n	Mean	SD
**Sex**	Male	214	27.9*	8.8
	Female	1077	31.9	7.8
**Age**	≤20	493	31.6	8.1
	21–24	643	31.0	8.0
	25–28	127	31.9	8.0
	≥29	88	30.1	7.9
**Dependents**	Yes	36	35.7	8.0
	No	1071	30.9*	8.0
**Works**	Yes	428	32.1	8.0
	No	892	30.8*	8.1
**Commuting time**	<30 minutes	1034	30.9*	8.1
	>30 minutes	259	32.7	7.4
**Scholarship**	Yes	719	31.9	7.9
	No	604	30.5*	8.1
**Academic variable**	**Category**	**n**	**Mean**	**SD**
**Course**	First	407	31.1	8.0
	Second	397	32.1	8.1
	Third	353	31.7	8.2
	Fourth	159	28.6*	7.1
**Disciplines enrolled for the second time**	Yes	259	32.5*	8.3
	No	979	30.8	7.8
**Practice contact**	Yes	1057	31.4	8.0
	No	261	30.8	8.0
**Academic activity**	**Perceived stress**	**n**	**Mean**	**SD**
**Master class**	None/Little	1067	30.4*	7.9
	Quite Much/Much	255	35.0	7.7
**Seminar**	None/Little	761	29.4*	7.9
	Quite Much/Much	555	33.7	7.5
**Problem-based learning**	None/Little	470	29.2*	7.9
	Quite Much/Much	773	32.6	7.8
**Laboratory/simulation**	None/Little	624	30.0*	8.2
	Quite Much/Much	686	32.3	7.8
**Clinical practices**	None/Little	581	29.7*	8.3
	Quite Much/Much	689	32.4	7.5
**Team work**	None/Little	624	29.5*	8.2
	Quite Much/Much	700	32.8	7.5
**Exams/tests**	None/Little	92	23.4*	8.2
	Quite Much/Much	1230	31.8	7.7
**Written work**	None/Little	467	28.2*	8.0
	Quite Much/Much	855	32.9	7.6
**Oral presentation**	None/Little	268	28.9*	8.9
	Quite Much/Much	1054	31.8	7.7

*: p < 0.005 Wilcoxon Rank Sum Test (Mann Whitney U test). Source: elaborated by the authors.

The previous tables can be contrasted with the regression analysis presented in Table 5, where those that have a p value with statistical significance are highlighted (*: p < 0.05).

**Table 5:** Multiple regression analysis of EES scale results – UFRO-Temuco (Chile), UdL-Lleida (Spain), URV-Tarragona-Tortosa (Spain), UdG-Girona (Spain), 2017-2018.

EES total	Coef.	Std. err.	t	P >|t|	[95% Conf. Interval]
Sex	–1.682	0.588	–2.86	0.004*	–2.836 –0.528
Dependents	3.691	1.749	2.11	0.035*	0.261 7.123
Works	–0.519	0.483	–1.07	0.283	–1.467 0.430
Stress level (SL) class classes	3.578	0.565	3.18	0.002*	0.400 1.691
Seminar SL	1.633	0.465	3.51	0.011*	0.263 2.052
PBL SL	1.157	0.456	2.54	0.005*	0.243 1.329
Practices SL	2.206	0.435	5.07	0.000*	1.352 3.060
Group work SL	1.755	0.440	3.99	0.000*	0.891 2.619
Exams SL	6.570	0.835	7.86	0.000*	4.930 8.209
Written work SL	2.221	0.466	4.76	0.000*	1.306 3.136
Age					
21–24	–0.267	0.479	–0.56	0.577	–1.206 0.672
25–28	–0.567	0.780	–0.73	0.467	–2.097 0.962
≥29	–2.004	0.994	–2.02	0.044*	–3.954 –0.053
Commuting time (30 minutes)	1.710	0.530	3.23	0.001*	0.670 2.750
Cons	–2.251	3.128	–0.72	0.472	–8.389 3.887

*: No. of observations 1088, Prob Model > F = 0.0000, R^2^= 0.25.

It should be noted that the relationship that “work” has on emotional exhaustion when analyzed individually is modified when the academic variables are introduced into the model, and is not significant in the multiple regression analysis. On the other hand, the categorized age behaves as a confounding variable on the relationship that the variable “having dependents” has on emotional exhaustion, fundamentally determined by the subgroup ≥29 years, making it significant, and shall be included in the model.

## DISCUSSION

Regarding the first objective, the sociodemographic variables, of the 1,368 participants who completed the questionnaire, the predominance was of the female sex, single people, without dependents or work, being consistent with another study that observed that these conditions seem to be critical when exhaustion emerged^(16)^. The significant differences evidenced between the Catalan and Chilean institutions, with the sociodemographic variables and the commuting time, could be attributed to the countries idiosyncrasy. With regard to the financing system, regardless of the type and financial aid, they constitute stress factors^(17)^, which, if prolonged over time, can lead to both personal and academic consequences^(2)^.

Regarding the second objective “Relate academic, sociodemographic variables and level of stress with emotional exhaustion among the students of the participating universities”, in a general way it could be mentioned that all those activities where the student is the protagonist of his own learning and assumes responsibility for their training process, including oral presentations, PBL, clinical simulation and clinical practices^(15,19,24–26)^, are stress factors that affect emotional exhaustion, which is consistent with the literature and confirms the hypothesis related to academic variables.

Exams are also stressful factors, which is consistent with what has been reported^(20,28)^. The significant difference observed between UFRO and the Catalan Universities could be attributed to the fact that the lower stress of Chilean students is the result of the use of problem-based learning from the first year and throughout their training process, being evaluated with instruments accordingly. Just as true, one shall keep in mind that besides exams, there is students’ exhaustion derived from the amount of academic homework^(11)^. Working in this modality means that stress also decreases when strategies such as seminars, group work, written work and, obviously, master classes are used, since the latter only meet the needs originated by problem-based learning^(20)^.

Regardless of the significant difference in the level of stress perceived in Laboratory/Simulation and clinical practices, this study, as well as that reported in the literature, shows that they cause a level of stress between moderate and high^(15,24–26)^. The difference found may be due to the fact that, at UFRO, the participants accessed, at that time, mainly clinical skills laboratories.

The stress perceived by students in academic activities, in each University, affirms that they correspond to academic and clinical factors^(6)^, which according to the literature can decrease academic performance, with eventual dropout and a direct relationship with clinical syndromes such as anxiety and depression^(3,4,6,7,20)^, and even increased substance use^(28)^.

With respect to the sociodemographic variables and their relationship with emotional exhaustion, in this study, as in others, women have a higher score, which may be due, according to some authors, to specific psychosocial traits^(13,14,16)^ which, particularly in this study, correspond to those who have dependents, work, have scholarships; or because, unlike men, they experience a higher degree of perceived stress in situations related to emotions^(15)^, which confirms the first hypothesis formulated for this study.

Regardless of whether the academic activities are centered on the student or the teacher, what was found as Quite Much/Much perceived stress in all of them makes the participants of all institutions have a medium level of emotional exhaustion, which can be attributed to the fact that the study plans have too much content, with excessive diligence constituting factors that affect learning^(21,27)^. In any case, the evidence found shall alert teachers to identify, in students, clinical syndromes such as anxiety and depression, self-esteem problems, and difficulty regulating emotions^(10,11)^.

As in another study, it was found that the overall EES score is higher in those who have taken disciplines for the second time^(16)^, which should be considered, since, according to the literature, students with higher levels of fatigue have few expectations of finishing their studies successfully and are also poorly prepared to face the world of work^(4)^.

Among the limitations, it should be mentioned that the self-reported instrument may give rise to some biased answers, since the participants may feel vulnerable when being consulted about private aspects of their life and this may even lead them not to answer all the items. On the other hand, the non- random sampling leads to poor generalizability of results, therefore limiting the results external validity.

The relevance of this study lies in the fact that the information collected will contribute to improving the teaching quality in the Nursing Career of the 4 institutions, given that having current and specific data will allow decision-making and proposals for improvement and/or strengthening of strategies that respond to the problem detected, and will prepare students to become aware of the challenges they have to assume. This research also contributes to other training centers for future nurses, since the academic and sociodemographic situations are similar.

## CONCLUSIONS

In all universities, regardless of the students’ different factors and characteristics, a medium level of emotional exhaustion was detected. The factor sex is a significant aspect, as well as sociodemographic factors such as added family responsibilities or the need for scholarships to finance studies. Moreover, the stress generated by teaching methodologies has a significant effect on the levels of emotional exhaustion at a global level. In addition, the characteristics of the training process itself which, in approximately 50%, takes place in clinical environments and in themselves are stressful and therefore lead to emotional exhaustion.

Given the results showing that the students of the nursing careers from the four universities have emotional exhaustion at a medium level, the institutions are forced to be on the alert and take preventive measures and promote healthy academic environments, with opportunities of psychosocial support.
